# Characterization of the phosphotransacetylase-acetate kinase pathway for ATP production in *Porphyromonas gingivalis*

**DOI:** 10.1080/20002297.2019.1588086

**Published:** 2019-04-04

**Authors:** Yasuo Yoshida, Mitsunari Sato, Takamasa Nonaka, Yoshiaki Hasegawa, Yuichiro Kezuka

**Affiliations:** a Department of Microbiology, School of Dentistry, Aichi Gakuin University, Nagoya, Japan; b Division of Structural Biology, Department of Pharmaceutical Sciences, School of Pharmacy, Iwate Medical University, Yahaba, Japan

**Keywords:** *Porphyromonas gingivalis*, phosphotransacetylase, acetate kinase, ATP, essential genes, crystal structure

## Abstract

Acetyl phosphate (AcP) is generally produced from acetyl coenzyme A by phosphotransacetylase (Pta), and subsequent reaction with ADP, catalyzed by acetate kinase (Ack), produces ATP. The mechanism of ATP production in *Porphyromonas gingivalis* is poorly understood. The aim of this study was to explore the molecular basis of the Pta-Ack pathway in this microorganism. Pta and Ack from *P. gingivalis* ATCC 33277 were enzymatically and structurally characterized. Structural and mutational analyses suggest that Pta is a dimer with two substrate-binding sites in each subunit. Ack is also dimeric, with a catalytic cleft in each subunit, and structural analysis indicates a dramatic domain motion that opens and closes the cleft during catalysis. ATP formation by Ack proceeds via a sequential mechanism. Reverse transcription-PCR analysis demonstrated that the *pta* (*PGN_1179*) and *ack* (*PGN_1178*) genes, tandemly located in the genome, are cotranscribed as an operon. Inactivation of *pta* or *ack* in *P. gingivalis* by homologous recombination was successful only when the inactivated gene was expressed *in trans*. Therefore, both *pta* and *ack* genes are essential for this microorganism. Insights into the Pta-Ack pathway reported herein would be helpful to understand the energy acquisition in *P. gingivalis*.

*Porphyromonas gingivalis*, a black-pigmented Gram-negative rod-type bacterium, is a keystone pathogen linked to the onset and progression of periodontitis [1,2]. Although periodontitis is localized to tissues surrounding teeth, it is associated with serious systemic conditions such as cardiovascular disease [3,4], diabetes [5], and rheumatoid arthritis [6].

The main routes for ATP and energy production in *P. gingivalis* remain to be fully elucidated. *P. gingivalis* is unable to ferment carbohydrates such as glucose, although small quantities of glucose monomers can be utilized for polymer biosynthesis [7]. Therefore, *P. gingivalis* relies on catabolism of amino acids to generate metabolic energy [8,9]. In many bacteria, the enzymes phosphotransacetylase (Pta, EC 2.3.1.8) and acetate kinase (Ack, EC 2.7.2.1) form a key pathway for production of ATP from excess acetyl coenzyme A (acetyl-CoA) [10]. Pta produces acetyl phosphate (AcP) from acetyl-CoA, and AcP is subsequently converted to acetate by Ack, with the concomitant production of ATP through substrate-level phosphorylation.

AcP functions as a signaling molecule that is capable of serving as a phosphate donor for two-component signal transduction systems, thereby connecting central metabolism with environment sensing and signal transduction [11,12]. Interestingly, inactivation of the Pta-Ack pathway in *Escherichia coli* results in biofilm formation that is quantitatively and architecturally distinct from that in parental strains [12–14]. However, disruption of the Pta-Ack pathway does not affect cell growth in many bacteria, including *E. coli* [15], *Streptococcus mutans* [16], *Streptococcus pneumoniae* [13], *Clostridium acetobutylycum* [17], *Clostridium tyrobutyricum* [18], and *Bacillus subtilis* [19]. Indeed, the presence of alternate pathways has been demonstrated for several bacterial, including *B. subtilis* [20], and a search of all available Ack sequences showed that more than 300 species possess multiple Ack enzymes [21]. By contrast, a recent study revealed the indispensable role of the Pta-Ack pathway in *Staphylococcus aureus* for maintaining energy and metabolic homeostasis during overflow metabolism [22].

Since *P. gingivalis* produces a large amount of short chain fatty acids, including acetate, propionate, and butyrate [23], it is postulated that the Pta-Ack pathway, which is associated with acetate production, is conserved in *P. gingivalis* [8,9] (Figure 1). Despite the importance of Pta and Ack in carbon cycling and energy metabolism in *P. gingivalis*, these enzymes have not been characterized in this microorganism, even though the coding genes have been assigned based on sequence homology [24,25]. In the current study, recombinant Pta (*Pg*Pta) and Ack (*Pg*Ack) from *P. gingivalis* ATCC 33277, encoded by *pta* (*PGN_1179*) and *ack* (*PGN*_*1178*), respectively, were prepared and enzymatically characterized. In addition, crystal structures were determined by X-ray crystallography. Based on the structures, several putative functional residues were identified and their contribution to catalytic activity was evaluated by site-directed mutagenesis. Furthermore, we examined the essentiality of *pta* and *ack* in *P. gingivalis*.

**Figure 1.**
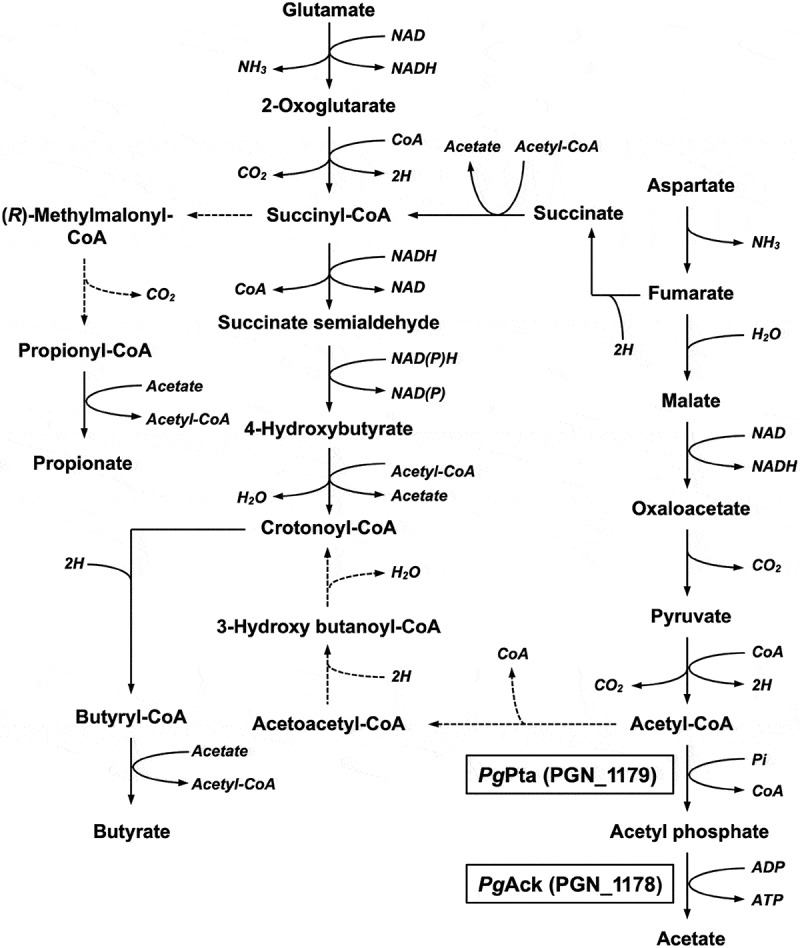
Proposed metabolic pathways for use of glutamate and aspartate in *P. gingivalis* based on previously described pathways [8,9,67,6^8^]. Broken lines indicate expected pathways that were not supported by the experimental evidence.

## Materials and methods

### Bacterial strains and growth conditions

All bacterial strains used in this study are listed in Table 1. *P. gingivalis* ATCC 33277 and its derivatives were grown in modified GAM broth (Nissui, Tokyo, Japan), or on Brucella HK agar (Kyokuto Pharmaceutical Industrial, Tokyo, Japan) supplemented with 5% rabbit blood at 37°C in an anaerobic atmosphere (80% N_2_, 10% H_2_, 10% CO_2_). Ampicillin or tetracycline was added to a final concentration of 10 µg/mL and 1 µg/mL, respectively, when needed. *E. coli* DH5α (Invitrogen, Carlsbad, CA) and BL21 (DE3) (Novagen, Madison, WI) strains were grown aerobically at 37°C in 2 × YT or LB medium (Thermo Fisher JP, Tokyo, Japan) supplemented with 100 µg/mL ampicillin, as required.

**Table 1.** Bacterial strains and plasmids used.

Strains and plasmids	Relevant characteristics	References
Strains *E. coli*		
DH5α	F^–^*end a hsdR17* (r_K_ ^–^ m_K_^+^) *supE44 thi-1 recA1 gyr-A96 ϕ80lacZ*M15	Invitrogen
BL21 (DE3)	F ^–^ *ompT hsdSB* (r_B_ ^–^ m_B_^–^) *gal dcm rne131*	Novagen
*P. gingivalis*		
ATCC 33277	Parent strain	ATCC
PGAGU1179P	*P. gingivalis* ATCC33277 harboring the *Pgpta* gene in pComp1179	This study
PGAGU1178P	*P. gingivalis* ATCC33277 harboring the *Pgack* gene in pComp1178	This study
PGAGU124	*P. gingivalis* ATCC33277 with the *cepA* cassette replacing *PGN_1180*	This study
PGAGU125	*P. gingivalis* PGAGU1179P with the *cepA* cassette replacing *pta*	This study
PGAGU126	*P. gingivalis* PGAGU1178P with the *cepA* cassette replacing *ack*	This study
PGAGU127	*P. gingivalis* ATCC33277 with the *cepA* cassette replacing *PGN_1177*	This study
Plasmids		
pMCL200	Cm^R^; cloning vector	[33]
pCEPA	Ap^R^ in *E. coli* and *P. gingivalis*; pCR-Blunt II TOPO (Thermo Fisher) containing *cepA* with its promoter and terminator	[32]
pKO1180-*cepA*	Cm^R^ and Ap^R^, pMCL200 derivative containing *cepA* flanked by the upstream and downstream regions of *PGN_1180*	This study
pKO1179-*cepA*	Cm^R^ and Ap^R^, pMCL200 derivative containing *cepA* flanked by the upstream and downstream regions of *pta*	This study
pKO1178-*cepA*	Cm^R^ and Ap^R^, pMCL200 derivative containing *cepA* flanked by the upstream and downstream regions of *ack*	This study
pKO1177-*cepA*	Cm^R^ and Ap^R^, pMCL200 derivative containing *cepA* flanked by the upstream and downstream regions of *PGN_1177*	This study
pTRAG402	Ap^R^ and Tc^R^ in *E. coli*, Tc^R^ in *P. gingivalis*; shuttle vector	[27]
pComp1179	Ap^R^ in *E. coli*, Tc^R^ in *P. gingivalis*; pTRA402 derivative containing *pta*	This study
pComp1178	Ap^R^ in *E. coli*, Tc^R^ in *P. gingivalis*; pTRA402 derivative containing *ack*	This study
pGEX-6P-1	Ap^R^; GST fusion expression vector	GE Healthcare
pET28a	Km^R^; (His)_6_ fusion expression vector	Merck
pPR1179-Gex	Ap^R^; pGEX-6P-1 derivative carrying *pta*	This study
pPR1179-R89A-Gex	Ap^R^; pGEX-6P-1 derivative carrying the R89A *Pg*Pta mutant	This study
pPR1179-R135A-Gex	Ap^R^; pGEX-6P-1 derivative carrying the R135A *Pg*Pta mutant	This study
pPR1179-D309A-Gex	Ap^R^; pGEX-6P-1 derivative carrying the D309A *Pg*Pta mutant	This study
pPR1179-S311A-Gex	Ap^R^; pGEX-6P-1 derivative carrying the S311A *Pg*Pta mutant	This study
pPR1179-R312A-Gex	Ap^R^; pGEX-6P-1 derivative carrying the R3212A *Pg*Pta mutant	This study
pPR1179-D318A-Gex	Ap^R^; pGEX-6P-1 derivative carrying the D318A *Pg*Pta mutant	This study
pET1178-Pet	Ap^R^; pET28a derivative carrying *Pgack*	This study
pET1178-R91A-Pet	Ap^R^; pET28a derivative carrying the R91A *Pg*Ack mutant	This study
pET1178-R241A-Pet	Ap^R^; pET28a derivative carrying the R241A *Pg*Ack mutant	This study
pET1178-E385A-Pet	Ap^R^; pET28a derivative carrying the E385A *Pg*Ack mutant	This study

### Expression of recombinant PgPta and PgAck

Recombinant *Pg*Pta and *Pg*Ack were overproduced using *E. coli* after cloning each coding region between appropriate restriction sites in pGEX-6P-1 (GE Healthcare Japan, Hino, Japan) and pET28a (Merck, Darmstadt, Germany), respectively. Primers used for cloning are listed in the Supplemental Table. *Pg*Pta has a glutathione *S*-transferase (GST)-tag cleavable by PreScission protease, whereas *Pg*Ack possesses an N-terminal (His)_6_-tag followed by a thrombin cleavage site. After verification by DNA sequencing, the resulting pPR1179-Gex and pET1178-Pet constructs (Table 1) were used to transform competent *E. coli* BL21 (DE3) cells. Transformants were cultivated at 37°C to an optical density at 600 nm of ~0.3, and isopropyl β-D-1-thiogalactopyranoside was added at a final concentration of 0.3 mM to induce expression of recombinant proteins. Culturing was continued at 37°C for ~3 h, and cells were harvested by centrifugation at 4°C.

Site-directed mutagenesis of *pta* and *ack* genes in *P. gingivalis* was performed using overlap extension PCR [26] or a KOD-Plus-Mutagenesis Kit (Toyobo, Osaka, Japan) with primers listed in the Supplemental Table, as previously described [27]. Overproduction of mutant enzymes was performed using the same procedure for wild-type (WT) enzyme described above.

### Purification of recombinant PgPta and PgAck

To purify recombinant *Pg*Pta, harvested cells were resuspended in phosphate-buffered saline (PBS) and lysed by ultrasonication on ice. The lysate was centrifuged at 30,000 × *g* for 1 h, and the GST fusion protein in the supernatant was absorbed onto an affinity matrix glutathione-Sepharose 4B column and cleaved with PreScission protease (GE Healthcare Japan) according to the manufacturer’s protocol. The tag-digested protein solution was loaded onto a MonoQ 10/100 GL column (GE Healthcare Japan) and eluted with a six column volume linear gradient of NaCl from 100 to 500 mM. Fractions containing recombinant *Pg*Pta were pooled, concentrated, and loaded onto a HiLoad 16/60 Superdex 200 pg column (GE Healthcare Japan) pre-equilibrated with 10 mM Tris/HCl pH 7.6 containing 10 mM NaCl.

For purification of recombinant *Pg*Ack, harvested cells were resuspended in 50 mM potassium phosphate (pH 8.0) containing 250 mM NaCl, and lysed by ultrasonication on ice. The lysate was centrifuged at 30,000 × *g* for 1 h, and recombinant *Pg*Ack was purified using TALON Superflow resin (GE Healthcare Japan) according to the manufacturer’s protocol. The (His)_6_-tag was cleaved by thrombin (Sigma-Aldrich, St. Louis, MO) according to the manufacturer’s instructions, and the tag-digested protein was further purified using MonoQ 10/100 GL and HiLoad 16/60 Superdex 200 pg columns as described above for *Pg*Pta.

Purified enzymes were stored at – 20°C after adding an equal volume of 80% glycerol. Protein concentrations were determined as described previously [28] and protein purity was assessed by SDS-PAGE and gel filtration chromatography using a Superdex 200 HR 10/30 column (GE Healthcare) at a flow rate of 0.3 mL/min in PBS. Molecular mass estimation of purified enzymes (240 µg/200 µL) was also performed by gel filtration chromatography using a calibrated column and protein standards (molecular weight range of 12,000–200,000) purchased from Sigma-Aldrich.

### Colorimetric enzyme assays

***i) PgPta activity.*** The ability of *Pg*Pta to produce AcP from acetyl-CoA was investigated by spectrophotometrically quantifying the consumption of acetyl-CoA during the reaction as previously described [29] with several minor modifications. Briefly, each reaction (100 µL) consisted of 100 mM Tris/phosphate (pH 9.0), 20 mM KCl, varying concentrations of acetyl-CoA (50–200 µM), and 1.8 ng/mL recombinant *Pg*Pta. After reactions were initiated by addition of enzyme and precisely incubated for 3 min at 37°C, samples were spectrophotometrically examined by measuring the absorbance at 233 nm (*A*_233_). The concentration of acetyl-CoA was calculated using a standard curve, and kinetic parameters of *Pg*Pta were calculated by fitting directly to initial rate vs. substrate concentration (*V* vs. *S*) curves based on the Michaelis-Menten equation. To analyze the optimal pH, reaction mixtures consisting of 50 mM Tris/phosphate buffer were adjusted to pH 6.0, 7.0, 8.0, 9.0, or 10.0. Data were obtained from three independent experiments.

***ii) PgAck activity.*** The activity of *Pg*Ack was determined by monitoring NADPH production as previously described [30] with several modifications. Reactions (150 µL) consisted of 50 mM Tris/HCl (pH 8.0), 2 mM dithiothreitol, 3.2 mM glucose, 1.3 mM NADP, 3.7 U/mL hexokinase (Sigma-Aldrich), 0.93 U/mL glucose-6-phosphate dehydrogenase (Sigma-Aldrich), 1.3 mM MgCl_2_, 67 ng/mL recombinant *Pg*Ack, varying concentrations of lithium potassium AcP (0.0667, 0.133, 0.267, and 0.667 mM), and ADP (0.267, 0.667, 1.33, and 2.67 mM). Reactions were initiated by the addition of substrate and incubation for 5 min at 37°C, and the amount of ATP in the reaction mixture was determined by spectrophotometrically quantifying NADPH at *A*_340_ using standard curves. Initial velocities of *Pg*Ack were determined at four fixed ADP concentrations at different AcP concentrations, and double reciprocal (Lineweaver-Burk) plots were obtained. Secondary plots of the *y* intercept (velocity reciprocal) vs. the inverse concentration of ADP were used to obtain *V*_max_ value and *K*_m_ value for ADP. Secondary plots of reciprocal slopes from Lineweaver-Burk plots vs. the inverse concentration of ADP provided *K*_m_/*V*_max_ values, which were used to calculate *K*_m_ values for AcP. Assay mixtures used for divalent metal ion screening contained 1.3 mM MgCl_2_, 1.3 mM ZnCl_2_, 1.3 mM CaCl_2_, or 1.3 mM EDTA instead of 1.3 mM MnCl_2_. To analyze the optimal pH, reaction mixtures consisted of 50 mM Tris/HCl buffer with the pH adjusted to 6.0, 7.0, 8.0, 9.0, or 10.0. Data were obtained from three independent experiments.

### Reverse transcription (RT)-PCR

RT-PCR analysis was carried out as previously described [31]. Briefly, total RNA was isolated from *P. gingivalis* using ISOGEN-LS reagent (Nippon Gene, Tokyo, Japan) in accordance with the manufacturer’s instructions. Total RNA samples were treated with RNase-free DNase (TaKaRa Bio, Kusatsu, Japan) to remove traces of chromosomal DNA. Total RNA was reverse-transcribed with random hexadeoxyribonucleotide primers (TaKaRa Bio) using PrimeScript Reverse Transcriptase (TaKaRa Bio) according to the manufacturer’s instructions. Gene-specific primers used for RT-PCR are listed in the Supplemental Table. Reaction mixtures without reverse transcriptase served as negative controls to evaluate the presence of contaminating genomic DNA in samples.

### Construction of gene-deficient mutants by allelic replacement

Mutant strains of *P. gingivalis* lacking *PGN_1180, pta, ack*, or *PGN_1177* genes were constructed by replacing each gene with a 2,123 bp cassette containing the ampicillin resistance gene, *cepA*, flanked by upstream and downstream DNA sequences targeting the *P. gingivalis* gene of interest. Each targeting sequence was separately amplified using *P. gingivalis* ATCC 33277 genomic DNA as a template, and *cepA* was amplified from pCEPA [32]. Primers used for PCR are listed in the Supplemental Table. Three amplicons were linked using an In-fusion HD Cloning kit (TaKaRa Bio) and subcloned into pMCL200 [33]. The construct was sequenced to rule out unintended base changes. Each linearized plasmid was electroporated into *P. gingivalis* ATCC 33277 and its derivatives as previously described [34]. Briefly, an overnight culture in modified GAM broth was diluted 1:5 in 5 ml of fresh modified GAM broth and incubated for 8 h at 37°C to obtain competent cells in log phase. The cells were collected, washed three times with 0.1 mM HEPES, washed once and resuspended with 10% glycerol, and then stored at – 80°C until use. The linearized plasmids (approximately 5 µg) were added to the competent cells and the mixtures were pulsed with a Gene Pulser apparatus (Bio-Rad Laboratories, Hercules, CA) at 2.5 kV with a time constant of 5 ms. The cell suspensions were immediately added to 10 ml of modified GAM broth prewarmed anaerobically, and then incubated anaerobically at 37°C for 24 h without antibiotics. Aliquots of the cell suspension were plated on Brucella HK agar with 5% rabbit blood containing appropriate antibiotics and incubated anaerobically for 10–15 days at 37°C. PCR analysis of genomic DNA from mutant strains was performed to confirm appropriate gene insertion in ampicillin-resistant mutant colonies.

Conditional mutant strains were also constructed from *P. gingivalis* ATCC 33277 using pComp1179 or pComp1178 (Table 1). Plasmids contained *pta* or *ack* inserted between *Bam*HI and *Sal*I sites in pTRAG402 [27] under the control of the strong *ragA* promotor [35].

### Structure determination of recombinant PgPta and PgAck

To perform crystallization experiments, each purified protein was concentrated to 10 mg/mL. Crystallization experiments were performed at 20°C using the hanging-drop vapor diffusion method. Purified protein solution (0.8 μL) was mixed with reservoir solution (0.8 μL) and the resulting droplet was equilibrated against 200 μL reservoir solution in a well. Crystals of *Pg*Pta were obtained using reservoir solution containing 20% (w/v) polyethylene glycol (PEG) 3350 and 0.2 M ammonium tartrate dibasic. Crystals of *Pg*Pta in complex with acetyl-CoA were prepared by cocrystallization using droplets containing 5 mM acetyl-CoA equilibrated against the same reservoir. Before crystallization of *Pg*Ack, magnesium chloride was added to the protein solution at a final concentration of 10 mM. *Pg*Ack was crystallized using reservoir solution containing 0.2 M lithium sulfate monohydrate, 0.1 M Tris/HCl (pH 8.5), and 24% (w/v) PEG 3350.

X-ray diffraction data were collected using beamline NE3A at the Photon Factory Advanced Ring (Ibaraki, Japan) at 95 K. Crystals of *Pg*Pta and *Pg*Ack were cryoprotected by soaking briefly in mother liquor containing 10% (v/v) 2-methyl-2,4-pentanediol and 30% (w/v) PEG 3350, respectively. Diffraction data were indexed, integrated, and scaled using DIALS [36] and SCALA [37] as implemented in XIA2 [38]. The structures of *Pg*Pta and *Pg*Ack were solved by molecular replacement using MOLREP [39]. Search models were generated using the homology modeling server SWISS-MODEL [40] based on the structure of Pta from *Methanosarcina thermophila* (*Mt*Pta) [Protein Data Bank (PDB) ID: 2AF4] [41] and Ack from *Thermotoga maritima* (PDB ID: 2IIR) for *Pg*Pta and *Pg*Ack, respectively. The resultant structures were progressively refined by fitting to the electron density maps using COOT [42]. Manual adjustment of structures was interspersed with refinement cycles using REFMAC5 [43]. Final statistics for X-ray diffraction data and crystallographic refinement are summarized in Table 2.

**Table 2.** Statistics for X-ray diffraction data and crystallographic refinement of *Pg*Pta and *Pg*Ack.

Enzyme		*Pg*Pta (PGN_1179)	*Pg*Ack (PGN_1178)
Form		Acetyl-CoA-bound	Substrate-free
Intensity statistics			
	Beamline	NE3A	NE3A
	Wavelength (Å)	1.0000	1.0000
	Space group	*P*2_1_2_1_2	*P*2_1_
	Cell dimensions *a*/*b*/*c*/β (Å, °)	95.69/130.02/52.60	82.77/98.47/102.77/91.64
	Resolution range (Å)	43.4–2.04 (2.07–2.04)*^a^*	53.4–1.94 (1.97–1.94)*^a^*
	*R*-merge (%)	9.2 (22.7)*^a^*	9.3 (24.0)*^a^*
	Completeness (%)	100.0 (99.1)*^a^*	99.3 (92.2)*^a^*
	*I*/σ	16.9 (5.5)*^a^*	11.9 (5.8)*^a^*
	Multiplicity	12.6 (11.4)*^a^*	6.5 (6.5)*^a^*
Refinement statistics			
	*R*-factor	0.203	0.189
	Free *R*-factor	0.237	0.219
	No. of subunits per asymmetric unit	2	4
	No. of water molecules	224	974
	No. of ligand molecules	2 ACO*^b^*	5 SO4*^b^*, 2 MPD*^b^*
	Average *B*-factors (Å) Protein/Waters/Ligands	22/24/63	18/23/42
	RMSD from ideal values Bond length (Å) Bond angle (°)	0.011 1.523	0.011 1.370
	Ramachandran plot*^c^* (%) Favored/Allowed/Outliers	97.9/2.1/0.0	99.0/1.0/0.0

*^a^* Values in parentheses are statistics for the highest resolution shell.

*^b^* ACO, Acetyl-CoA; SO4, sulfate ion; MPD, 2-methyl-2,4-pentanediol.

*^c^* Calculated using the program RAMPAGE [66].

### Statistical analyses

Differences between groups were assessed by one-way analysis of variance and Student-Newman-Keuls multiple comparison post-tests or Student’s *t*-tests. Differences were considered statistically significant at p < 0.01.

### Accession numbers

Atomic coordinates and structure factors for *Pg*Pta in complex with acetyl-CoA, and *Pg*Ack, have been deposited at the PDB under accession codes 6IOX and 6IOY, respectively.

## Results

### Purification and enzymatic characterization of recombinant PgPta and PgAck proteins

**Figure 2.**
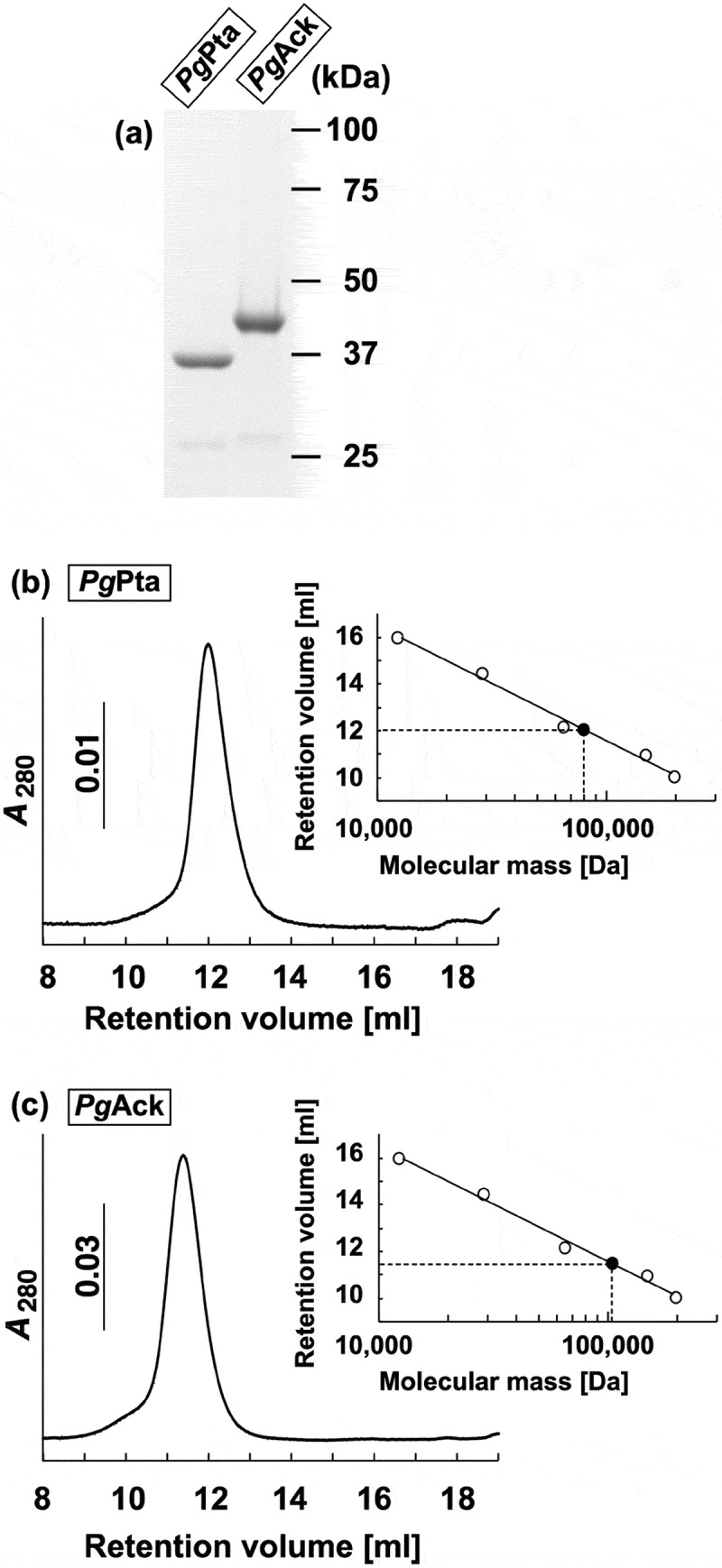
Purification and gel filtration analysis of recombinant *Pg*Pta and *Pg*Ack proteins. (a) SDS-PAGE analysis of recombinant *Pg*Pta and *Pg*Ack proteins. Samples (~5 μg) were subjected to SDS-PAGE and visualized by Coomassie Brilliant Blue staining. The positions of molecular mass markers (in kDa) are shown. (b and c) Gel filtration chromatography analysis of the molecular mass of *Pg*Pta (b) and *Pg*Ack (c). A calibration curve was constructed using protein standards (shown as open circles in the inset). Purified *Pg*Pta and *Pg*Ack are represented by closed circles.

The final step in the production of ATP from acetyl-CoA in *P. gingivalis* is believed to be carried out by the enzymes associated with the Pta-Ack pathway (Figure 1). A homology search revealed that the amino acid sequences of *Pg*Pta (PGN_1179) and *Pg*Ack (PGN_1178) from *P. gingivalis* share 45% and 47% identity, respectively, with *Mt*Pta and *Mt*Ack from *M. thermophila* (Supplemental Figures S1 and S2), which are well-characterized [44–46]. To enzymatically characterize *Pg*Pta and *Pg*Ack, recombinant proteins were expressed in *E. coli* and purified, and homogeneity was confirmed by SDS-PAGE (Figure 2(a)). The molecular masses of denatured *Pg*Pta and *Pg*Ack were in agreement with the predicted values (35.8 kDa and 43.2 kDa, respectively). In gel filtration chromatography experiments, *Pg*Pta and *Pg*Ack eluted as single peaks at retention volumes corresponding to molecular masses of 80.5 and 108 kDa, respectively (Figures 2(b) and 2(c)), suggesting that *Pg*Pta is a homodimer in solution, and *Pg*Ack is a homodimer or homotrimer.

**Figure 3.**
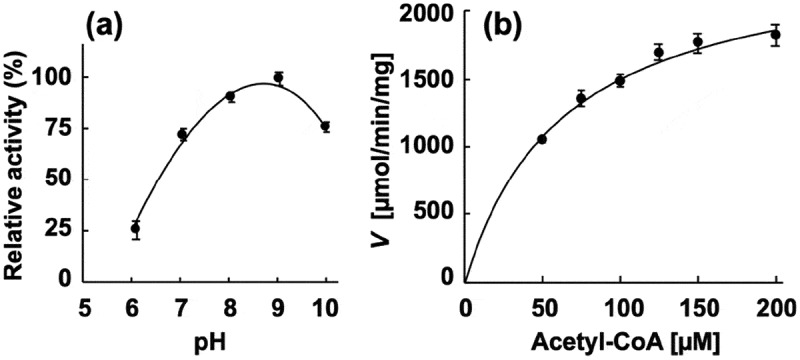
Enzymatic characterization of recombinant *Pg*Pta protein. (a) Effect of pH on AcP production by *Pg*Pta. The decrease in acetyl-CoA during incubation of recombinant *Pg*Pta with acetyl-CoA at pH 6.0–10.0 was spectrophotometrically quantified by measuring the absorbance at 233 nm (*A*_233_). Enzyme activities are indicated relative to the activity at pH 9.0. (b) Steady-state kinetic analysis of *Pg*Pta. Reaction mixtures contained 100 mM Tris/phosphate buffer (pH 9.0), 20 mM KCl, 1.8 ng/mL recombinant *Pg*Pta, and 50, 75, 100, 125, 150, or 200 µM acetyl-CoA. Averages from three independent experiments are presented.

**Figure 4.**
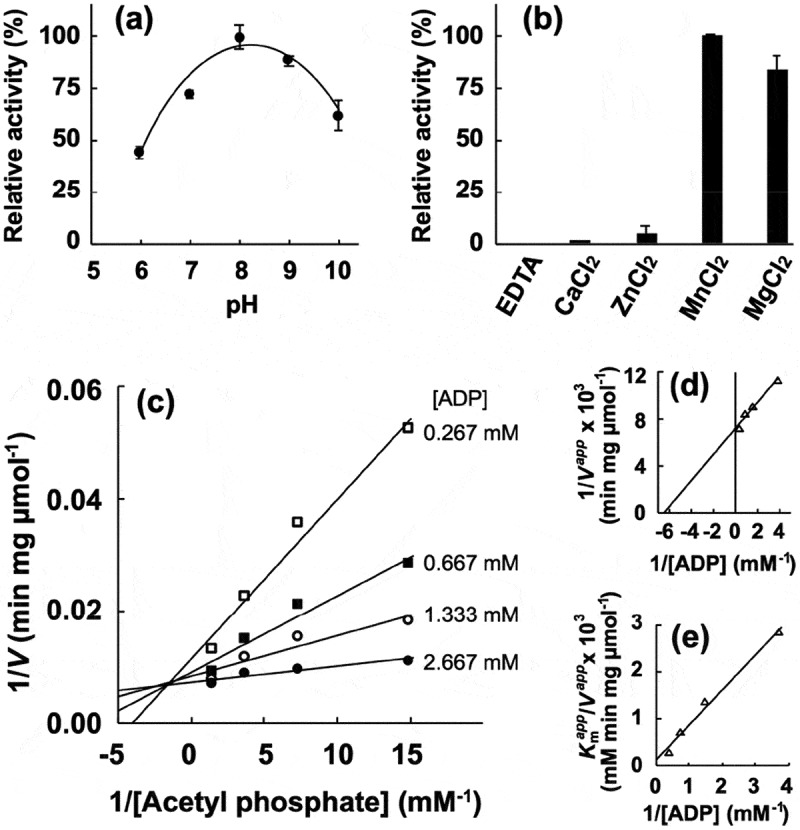
Enzymatic characterization of recombinant *Pg*Ack protein. (a) Effect of pH on production of ATP by *Pg*AcK from 0.3 mM AcP and 0.3 mM ADP. Enzyme activities are indicated relative to the activity at pH 8.0. (b) Effect of divalent metal ions on ATP production by *Pg*AcK from 0.3 mM AcP and 0.3 mM ADP. Enzyme activities at pH 8.0 are indicated relative to that in the presence of 1.3 mM MnCl_2_. (c) Steady-state kinetic analysis of *Pg*Ack using double reciprocal plots from initial velocity measurements determined in 50 mM Tris/HCl buffer (pH 8.0) at 37°C. Various concentrations of AcP (0.0667, 0.133, 0.267, and 0.667 mM) were tested for every fixed ADP concentration (0.267, 0.667, 1.33, and 2.67 mM). Averages from three independent experiments are presented. (d) Secondary plots of the *y* intercept (velocity reciprocal) vs. the inverse concentration of ADP. (e) Secondary plots of reciprocal slopes from panel (a) vs. the inverse concentration of ADP.

Next, the enzymatic activity of the recombinant proteins was characterized. The phosphotransacetylase activity of recombinant *Pg*Pta was evaluated by spectrophotometrically quantifying acetyl-CoA consumed during the reaction. The maximum activity of recombinant *Pg*Pta was observed at pH 9.0 (Figure 3(a)). Kinetic parameters of *Pg*Pta were determined using the Michaelis-Menten equation (Tables 3 and 3(b)), yielding *k*_cat_ and *K*_m_ values of 1,487 ± 63 s^–1^ and 63.8 ± 7.5 μM, respectively. Meanwhile, the acetate kinase activity of *Pg*Ack was determined by quantifying ATP produced from ADP and AcP. The optimal pH of *Pg*Ack was pH 8.0 (Figure 4(a)). Among the divalent metal ions tested (Mn^2+^, Mg^2+^, Zn^2+^, and Ca^2+^), Mn^2+^ and Mg^2+^ supported the high *Pg*Ack activity (Figure 4(b)). The activity of *Pg*Ack in the presence of Mg^2+^ was ~80% of that in the presence of Mn^2+^. No detectable activity was observed in the presence of EDTA. Initial reaction velocities of *Pg*Ack were determined at four fixed ADP concentrations at different AcP concentrations, and Lineweaver-Burk (double reciprocal) plots revealed a clear intersecting line pattern (Figure 4(c)), indicating that *Pg*Ack acts via a sequential mechanism [45]. The *V*_max_ (145 ± 1 μmol/min/mg) and *K*_m_ values for ADP (188 ± 22 μM) and AcP (22.2 ± 0.2 μM) were estimated from secondary plots (Figures 4(d) and 4(e)). The kinetic parameters are summarized in Table 3.

**Table 3.** Kinetic parameters for reactions catalyzed by recombinant *Pg*Pta and *Pg*Ack.

Enzyme	Substrate	*K*_m_ (µM)	*V*_max_ (µmol/min/mg)	*k*_cat_ (s^–1^)
*Pg*Pta	Acetyl-CoA	63.8 ± 7.5	2,471 ± 105	1,487 ± 63
*Pg*Ack	ADP	188 ± 22	145 ± 1	105 ± 1
	AcP	22.2 ± 0.2

### Structure of PgPta and enzymatic activity of its mutants

**Figure 5.**
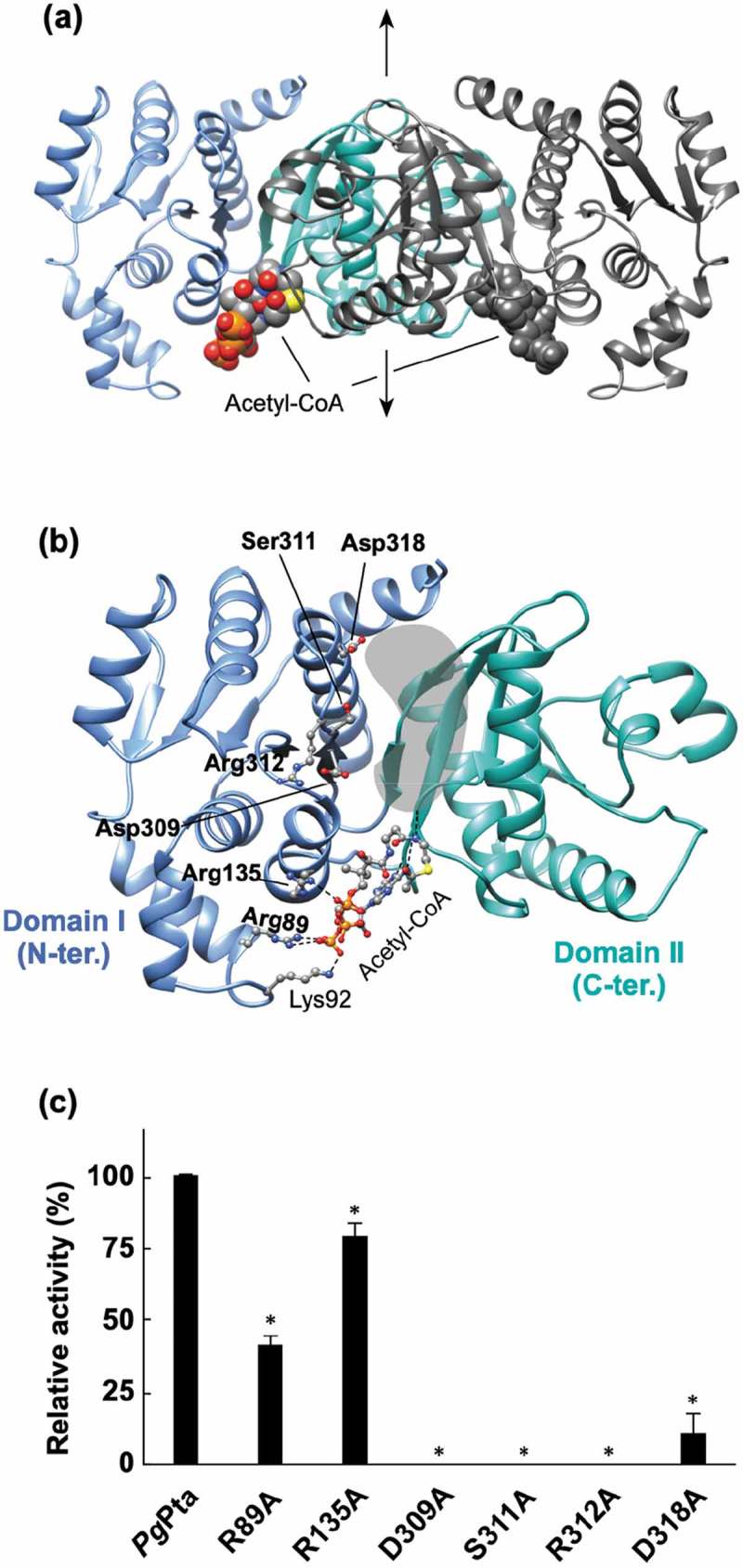
Structural analysis of *Pg*Pta. (a) The dimeric structure of *Pg*Pta in complex with acetyl-CoA. The two domains of one subunit of the dimer are colored light blue (domain I) and green (domain II), and both domains of the other subunit are colored dark gray. Bound acetyl-CoA molecules are shown as spheres. The 2-fold axis is indicated by arrows. (b) The subunit structure of *Pg*Pta viewed in the same orientation as (a). Residues mutated in this study (labeled with bold letters) and bound to acetyl-CoA (Lys92) are shown in ball-and-stick representation. The gray-colored region at the domain interface is a putative active site. (c) Enzyme activity of WT and mutant *Pg*Pta proteins. Initial acetyl-CoA consumption velocities were measured, and enzyme activities are indicated relative to that of WT *Pg*Pta. Data are presented as means ± standard deviation of three independent experiments (*p < 0.01).

The structure of *Pg*Pta in complex with acetyl-CoA was determined at 2.0 Å resolution (Figure 5(a) and Table 2). The asymmetric unit of the *P*2_1_2_1_2 cell contains one dimer. The subunit is an α/β protein composed of two α/β domains (Figure 5(b)); N-terminal domain I (residues 1–145 and 302–336) and C-terminal domain II (residues146–301), based on the previous definition [44]. Domain II is responsible for dimerization (Figure 5(a)). The N- and C-terminal domains are positioned side by side, and the domain arrangement results in the formation of a cleft with a putative active site at the interface.

Three distinct conformations of the subunit structure have been reported for *Mt*Pta [44]; a change in the position of domain I relative to domain II results in distinct open, closed, and partially closed conformations. Structural comparison of the subunits of *Pg*Pta in complex with acetyl-CoA with those of *Mt*Pta showed that both subunits of *Pg*Pta are in the open conformation, with a root mean square deviation (rmsd) of 0.89 Å (314 Cα atoms) and 0.90 Å (318 Cα atoms) for subunits A and B, respectively. However, the extent of the conformational change observed in *Pg*Pta is much less than that in *Pg*Ack (described below).

Structural analysis revealed an acetyl-CoA molecule bound to each subunit (Figure 5(a)), with seven direct hydrogen bonds or electrostatic interactions between the bound acetyl-CoA and subunit B (Figure 5(b)). The adenine base is recognized by two hydrogen bonds with the main-chain amide and carbonyl groups of Val150. The 3´-phosphate interacts with the side chains of Arg89 and Lys92 in subunit B. Arg135 forms an electrostatic interaction with the β-phosphate. The second amide nitrogen is hydrogen-bonded to the carbonyl group of Ala175. The interactions provided from Lys92 and Ala175 are missing in subunit A due to differences in χ angles of side chains and/or the bound acetyl-CoA. This results in higher *B*-factor values for acetyl-CoA in subunit A (74 Å^2^) than subunit B (52 Å^2^).

Six amino acid residues (Arg89, Arg135, Asp309, Ser311, Arg312, and Asp318) positioned at the cleft and the acetyl-CoA binding site were mutated and recombinant proteins were prepared (Supplemental Figure S3) to investigate their contributions to catalytic activity. All the prepared mutants except D318A gave a single peak in the gel filtration (Supplemental Figure S3), indicating the mutations did not affect the dimerization. The D318A mutation displayed a broad biphasic pattern. However, it seems that more than half of the D318A mutant is still dimer. The crystal structure clearly shows that Arg89 and Arg135 interact with the 3´-phosphate and β-phosphate of acetyl-CoA, respectively (Figure 5(b)). Replacement of Arg89 and Arg135 resulted in a 58% and 21% decrease in specific activity, respectively (Figure 5(c)). The other four mutated residues are located at the cleft, not in contact with Arg89 and Arg135. The polypeptide region in which Asp309, Ser311, and Arg312 are located forms part of the side of the cleft on the domain I side, with Ser311 and Arg312 positioned at the edge of the cleft, and Asp309 located near the bottom of the cleft. Mutation of these residues resulted in the complete loss of enzyme activity. Finally, Asp318 is located at the edge of the cleft on the opposite side from the observed acetyl-CoA binding site. The D318A mutant displayed only 11% of the specific activity of the WT enzyme.

### Structure of PgAck and enzymatic activity of its mutants

**Figure 6.**
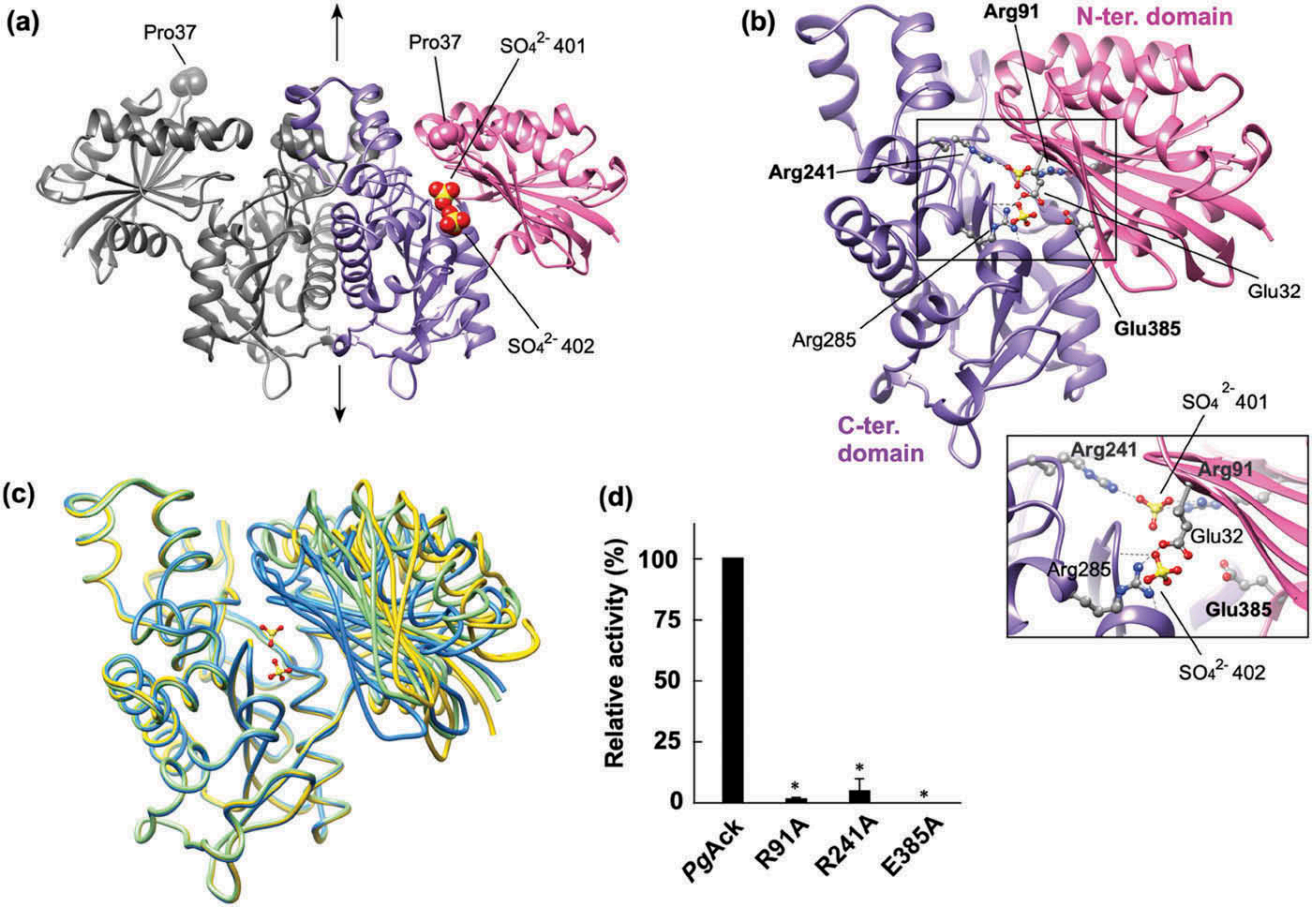
Structural analysis of *Pg*Ack. (a) The dimeric structure of *Pg*Ack (shown using subunits C and D). The two domains in subunit D are colored pink (N-terminal domain) and purple (C-terminal domain), and both domains of subunit C are colored dark gray. The two sulfate ions (SO_4_^2-^ 401 and 402) bound in the cleft in subunit D, and Pro37 at the edge of the cleft in both subunits, are shown as spheres. The 2-fold axis is indicated by arrows. (b) The subunit structure of *Pg*Ack viewed in the same orientation as (a). Residues mutated in this study (labeled with bold letters), with hydrogen-bonding interactions between the domains, and with bound sulfate ions are shown in ball-and-stick representation. Hydrogen bonds are indicated by dashed lines. An enlarged view of the active site is shown in the inset. (c) Superposition of *Pg*Ack subunits using Cα atoms of the C-terminal domain (rmsd <0.35 Å, ~234 Cα atoms). The position of the N-terminal domain relative to the C-terminal domain is different among the subunits. Subunits A, C, and D are colored yellow, light green, and blue, respectively. Although subunit B is slightly more similar in conformation to subunit A, it is not shown to simplify the figure. The two sulfate ions bound to subunit D are also illustrated. (d) Enzyme activity of WT and mutant *Pg*Ack enzymes. Initial ATP production velocities were measured, and enzyme activities are indicated relative to that of WT *Pg*Ack. Data are presented as means ± standard deviation of three independent experiments (*p < 0.01).

The structure of *Pg*Ack was refined at 1.9 Å resolution (Table 2). Four subunits (A, B, C, and D) are present in the asymmetric unit of the *P*2_1_ cell. Structural analysis by the PISA server [47] was performed to identify potential subunit interfaces and evaluate their significance for multimer formation. Interfaces between subunits A and B, and subunits C and D (interface area of ~3000 Å^2^) received significantly higher scores than others (data not shown), suggesting that *Pg*Ack forms a homodimer (Figure 6(a)), with two homodimers present in the asymmetric unit. This implies a dimeric state in solution, consistent with the ambiguous dimer or trimer predicted by gel filtration chromatography (Figure 2(b)). The structure of the *Pg*Ack subunit is composed of two α/β domains; the N-terminal domain (residues 1–149 and 384–398) and the C-terminal domain (residues 150–383, Figure 6(b)). The secondary structural elements of *Pg*Ack are well conserved with those of *Mt*Ack [48]. The C-terminal domain of each subunit is responsible for dimerization, and the dimer is related by the 2-fold symmetry axis (Figure 6(a)). A putative catalytic cleft is formed between the domains.

Comparison of the four subunits in the asymmetric unit revealed distinct conformations, with rmsd values ranging from 0.81 to 5.19 Å using all 398 Cα atoms (Figure 6(c)). The structure of the C-terminal domain is almost invariant among the subunits, and the positions of the N-terminal domain relative to the C-terminal domain are different, leading to opening and closing of the cleft (Figure 6(c)). The cleft in subunit D is the most closed, with two sulfate ions bound, presumably derived from lithium sulfate in the mother liquor. Compared with subunit D, the cleft in the other subunits is more open to varying extents and contains no bound ligands. Pro37 at the edge of the cleft is shifted 17.6, 14.0, and 11.7 Å in subunits A, B, and C, respectively, from the corresponding position in subunit D. The degree of opening is ordered subunits A, B, C, and D (highest to lowest). Enzyme activity assays revealed that Mg^2+^ or Mn^2+^ is required for catalytic activity of *Pg*Ack (Figure 4(b)). Interestingly, although we crystallized *Pg*Ack in the presence of Mg^2+^ (and Mn^2+^, data not shown), electron density corresponding to a divalent metal cation was not observed.

We performed a mutational analysis of three residues positioned at the cleft; based on the structure of *Pg*Ack and previous mutational studies on *Mt*Ack [46,49–51], and we selected Arg91, Arg241, and Glu385 for mutation. Arg91 and Arg241 interact with SO_4_^2-^ 401 in the crystal structure (Figure 6(b)), while Glu385 is positioned ~7 Å away from both sulfate ions. Following preparation of the mutant proteins (Supplemental Figure S3), enzymatic activity was compared (Figure 6(d)). All three mutations dramatically decreased the specific activity; R91A and R241A exhibited only 0.6% and 3.9% of the activity of the WT enzyme, and E385A displayed no detectable activity.

Gel filtration analysis of the mutants showed that each mutant protein mostly forms homodimer in common with the WT enzyme (Supplemental Figure S3). However, the peaks of E385A and R91A had obvious front shoulders with molecular mass of 217 kDa, which corresponds to almost the double of the estimated mass of the dimer (108 kDa). This result indicated that approximately 20% and 40% of the E385A and R91A mutants, respectively, appeared to be tetramer.

### Transcriptional analysis of Pta and Ack genes in P. gingivalis

**Figure 7.**
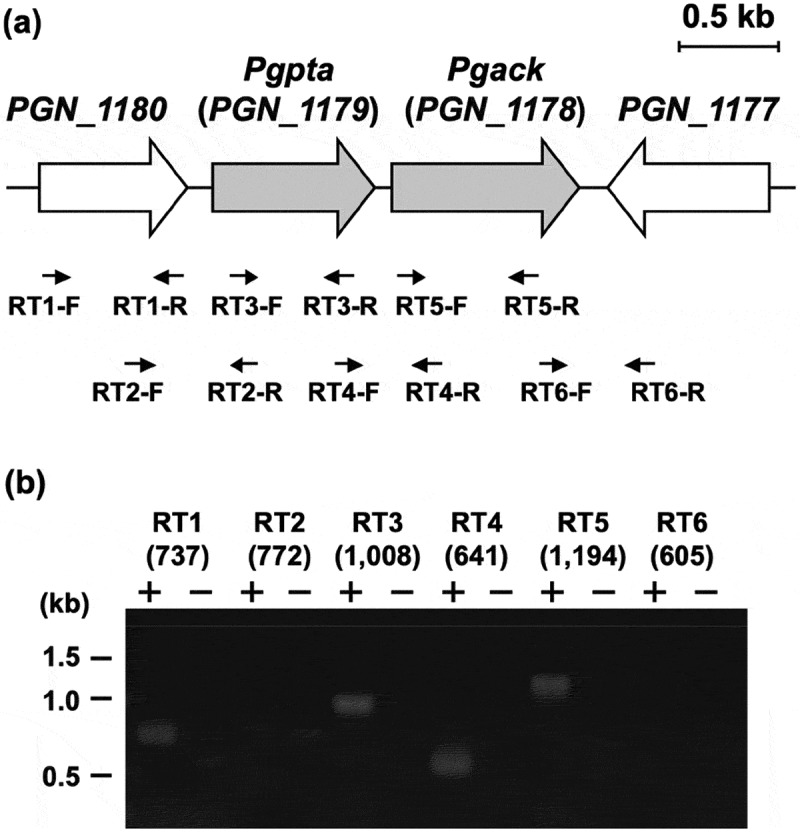
Genetic organization and transcriptional analysis of the *pta-ack* region of *P. gingivalis* ATCC 33277. (a) Gene arrangement within the *pta-ack* region of *P. gingivalis* ATCC 33277 chromosomal DNA. Large and small arrows indicate open reading frames and RT-PCR primers, respectively. (b) RT-PCR analysis of *pta* and *ack* gene expression. PCR amplicons were separated by 0.8% agarose gel electrophoresis. The predicted size (in bp) of each PCR amplicon is shown in parentheses. Lanes marked (+) or (**–**) indicate standard RT-PCR reactions or negative controls without cDNA, respectively. The positions of DNA size standards (in kb) are indicated on the left.

Transcriptional regulation of the *pta* and *ack* regions in *P. gingivalis* was characterized by RT-PCR (Figure 7). DNA fragments with expected length were effectively amplified using the primers (Supplemental Table) and the genomic DNA from *P. gingivalis* (Supplemental Figure S4). Transcripts corresponding to the regions spanning the borders of *pta* and *ack* were detectable, demonstrating that these genes are cotranscribed in this microorganism. PCR fragments corresponding to the regions spanning the border of *PGN_1180* and *pta* were not amplified. The function of the PGN_1180 protein is unknown. By contrast, the gene (*PGN_1177*) immediately downstream from *ack* was oppositely transcribed. Thus, only *pta* and *ack* were transcribed together as an operon. No significant products were amplified using cDNA prepared without reverse transcriptase, indicating that the RT-PCR products were not derived from contaminating chromosomal DNA (Figure 7).

## Both pta and ack genes are essential in P.gingivalis

To examine the physiological roles of *pta* and *ack* genes in *P. gingivalis* ATCC 33277, we attempted to construct chromosomal knockout mutants of each gene using a standard allelic replacement procedure that involves the insertion of an ampicillin resistance gene into the target gene (Supplemental Figure S5). However, attempts to obtain mutant *P. gingivalis* strains in which *pta* or *ack* was inactivated were unsuccessful (Table 4). Furthermore, when an erythromycin resistance cassette was used instead of an ampicillin resistance gene, no null mutants were obtainable (data not shown). By contrast, using the same procedure, it was possible to obtain null mutants of both *PGN_1180* and the *PGN_1177* genes, which are located upstream from *pta* and downstream from *ack*, respectively (Table 4).

**Table 4.** *P. gingivalis* transformation efficiency.

Recipient strain	Gene targeted for inactivation	Transformation efficiency (no. of transformants/µg DNA)
ATCC 33277	*PGN_1180*	19.3 ± 5.18
ATCC 33277	*pta*	n.d. *^a^*
ATCC 33277	*ack*	n.d. *^a^*
ATCC 33277	*PGN_1177*	33.2 ± 11.8
ATCC 33277 harboring *pta* in the plasmid	*pta*	10.9 ± 3.13
ATCC 33277 harboring *ack* in the plasmid	*ack*	15.7 ± 2.46

*^a^* Not detected.

Next, we attempted to construct chromosomal knockout mutants of *pta* and *ack* genes in the *P. gingivalis* ATCC 33277 background using pComp1179 and pComp1178, respectively, (Table 1) for expression of the respective genes in *P. gingivalis* under the control of the *ragA* promotor [27]. The introduction of *pta* and *ack* genes *in trans* led to disruption of the corresponding genes in the chromosomal DNA of *P. gingivalis*, and the transformation efficiencies for *pta-* and *ack*-deficient mutants were comparable with those for *PGN_1180*- and *PGN_1177*-deficient mutants (Table 4). The growth of all the mutant strains was similar to that of the parent strain (Supplemental Figure S6),

## Discussion

Knowledge of cell physiology and the conversion of nutrients to ATP, the universal molecular currency of energy transfer, is indispensable for understanding how bacteria survive, grow, reproduce, and express virulence factors. However, ATP production in *P. gingivalis* remains poorly understood. Indeed, the present study represents the first characterization of the Pta-Ack pathway associated with ATP production in *P. gingivalis*.

The *k*_cat_ value of *Pg*Pta (1,487 ± 63 s^–1^) is comparable to that of *Mt*Pta (1,500 ± 30 s^–1^) [52] and is more than 10-fold higher than those of other Pta orthologues [53,54]. By contrast, the *K*_m_ of *Pg*Pta (63.8 ± 7.5 μM) for acetyl-CoA is within the range reported for other Pta orthologues (8.6 to 600 μM) [53–56]. The pH optimum of *Pg*Pta was 9.0, which is slightly higher than the pH 8.0 reported previously for other Pta orthologues [29,54].

Structural analysis of *Pg*Pta revealed two acetyl-CoA molecules bound to the dimer (Figure 5(a)), and the acetyl-CoA binding site in each subunit is unique and identical. In *Mt*Pta, there are two CoA binding sites (1 and 2) in each subunit with different affinities [41]. Replacement of Ser309 and Arg310 located at binding site 1 in *Mt*Pta greatly affected the *k*_cat_ value [52], whereas mutation of Arg87 and Arg133 positioned at site 2 mainly impacted the *K*_m_ value [57]. Given the number of interactions between the bound CoA and each site, CoA at site 1 is thought to be more tightly bound to *Mt*Pta than CoA at site 2. Therefore, in *Mt*Pta, site 1 (the high-affinity site) is suggested to be the active site [41]. Site-directed mutagenesis of Arg89 and Arg135 in site 2 of *Pg*Pta (Arg87 and Arg133 in *Mt*Pta) had moderate effects on specific activity (Figure 5(c)), indicating that they are also not essential for catalysis. By contrast, mutation of residues at site 1 (D309A, S311A, R312A, and D318A) completely or dramatically decreased the catalytic activity. These findings suggest that site 1 was also the probable active site in *Pg*Pta. Contrary to predictions, acetyl-CoA molecules were bound only to site 2 in both subunits of *Pg*Pta (Figures 5(a) and 5(b)), even though crystal packing of *Pg*Pta does not appear to interfere with binding of acetyl-CoA to site 1. Since interference of the binding of acetyl-CoA to site 1 of *Pg*Pta has not been observed in solution, such contradictory findings might be due to crystallization artifacts. Dynamic aspects, such as domain motion, can affect substrate binding and are difficult to estimate from the static picture revealed by a crystal structure. Further analyses to investigate the binding of acetyl-CoA to *Pg*Pta are therefore required.

The pH optimum for *Pg*Ack (pH 8.0) is consistent with that of most other Ack enzymes reported to date [21,53]. Under the conditions tested, *Pg*Ack displayed highest activity in the presence of Mn^2+^, and was less efficient with Mg^2+^. Similarly, other Ack enzymes also function optimally with Mn^2+^ or Mg^2+^ [58]. The reaction velocities of *Pg*Ack exhibited conventional Michaelis-Menten kinetics, consistent with most other Ack enzymes [53]. Double reciprocal plots (Figure 4(c)) indicate that ATP formation by *Pg*Ack proceeds via a sequential mechanism.

There are four crystallographically independent *Pg*Ack subunits in the asymmetric unit with distinct conformations, corresponding to the degree of opening and closing of the catalytic cleft (Figure 6(c)). Such conformational changes are a common feature of the acetate and sugar kinase/Hsc70/actin (ASKHA) superfamily [59]. Structural analysis of *Mt*Ack with transition state analogs ADP, AlF_3_ (a mimic of the meta-phosphate transition state), and acetate strongly suggest that catalysis proceeds via a direct in-line phosphoryl transfer mechanism [51]. ADP, AlF_3_, and acetate are aligned in a linear array in the cleft in *Mt*Ack, and the catalytic cleft is wide open to accommodate all three ligands. By contrast, the cleft of subunit D is almost closed in the *Pg*Ack structure determined without any ligands such as ADP, AlF_3_, and acetate. This structure likely represents complete cleft closure because there is a direct contact between residues Glu32 and Arg285 positioned on opposite sides of the interface of the two domains (Figure 6(b)). Comparison of the *Mt*Ack subunit with bound transition state analogs and subunit D of *Pg*Ack reveals a shift in the N-terminal domain of 22 Å (Cα-Cα distance between residue 37 of the two enzymes) upon cleft closure. This estimated value is larger than expected (≤15 Å) for *Mt*Ack and propionate kinase, other members of the ASKHA superfamily [60]. Therefore, these results strongly indicate a dramatic domain motion in *Pg*Ack during catalysis via a direct in-line phosphoryl transfer mechanism.

There are several conserved residues at the active site of Ack enzymes [30], and their roles and contributions to enzymatic activity and/or substrate binding have been extensively examined for *Mt*Ack [46,49,50,60,61]. Arg91 and Arg241 in *Mt*Ack interact with the carboxyl group of acetate and are catalytically essential; R91A and R241A mutants of *Mt*Ack displayed *k*_cat_ values only 0.3 and 0.4% that of the WT enzyme, respectively [51]. The specific activities of *Pg*Ack mutants R91A and R214A showed a similar trend to the corresponding *Mt*Ack mutants, indicating their importance in catalysis. The E385A mutant of *Pg*Ack lost all enzyme activity (Figure 6(d)). The equivalent Glu384 in *Mt*Ack is believed to be responsible for divalent metal cation binding [46,60]. As shown in Figure 4(b), *Pg*Ack favors Mn^2+^ or Mg^2+^, either of which is indispensable for catalysis. Therefore, Glu385 in *Pg*Ack is a candidate metal-binding residue. As the result of gel filtration, the E385A and R91A mutants are like to be partly tetramer. Although it is unclear if the possible oligomeric transition affects the catalytic activity, a large proportion (60–80%) of the R91A and E385A mutants are present as dimer like the WT enzyme. Therefore, the no or little activity of the mutants would be mostly due to the single amino acid mutation.

Bacterial genes are often organized in operons with regulatory units that control genes/proteins with associated functions. The RT-PCR analysis demonstrated that *ack* and *pta* genes constitute an operon in *P. gingivalis* (Figure 7), as has also been shown for homologous genes in several bacteria including *E. coli* [15] and *Corynebacterium glutamicum* [62]. Transcription of the *PGN_1180* gene is independent from *pta* and *ack*. The PGN_1180 protein, the function of which has not been assigned, is therefore presumably not associated with ATP production.

Our experiments using conditional mutant strains clearly revealed that both *pta* and *ack* are essential genes in *P. gingivalis*, at least under the growth conditions tested (Table 4). A previous experiment using a transposon insertion mutant library demonstrated that 463 genes, including *pta* and *ack*, are essential for growth of *P. gingivalis* ATCC 33277 *in vitro* [63], whereas another independent experiment using the same bacterial strain and a similar method did not identify these genes as essential [64]. Of the 459 genes found to be essential by Klein et al. [63], only 281 genes (61%) were predicted to be essential by Hutcherson et al. [64]. It should be noted that the essentiality of genes can change depending on the growth conditions.

All living cells rapidly turn over ATP to satisfy their energy demands, and consequently ATP must be produced from ADP in a process requiring energy. In general, ATP is biosynthesized via substrate-level phosphorylation by enzymes in the cytoplasm, or through electron transfer by ATPases located on mitochondrial (eukaryotes) or cytoplasmic (prokaryotes) membranes. Since ATP production and substrate-level phosphorylation processes are directly coupled, the reactions necessarily liberate the amount of energy required to phosphorylate ADP [65]. Coupling to the phosphorylation of ADP is limited to a small number of molecules for which it is thermodynamically feasible: 1,3-bisphosphoglycerate, succinyl-CoA, phosphoenolpyruvate, arginine phosphate, carbamoyl phosphate, creatine phosphate, butyryl phosphate, and AcP, catalyzed by phosphoglycerate kinase, succinyl-CoA synthetase, pyruvate kinase, arginine kinase, carbamate kinase, creatine kinase, butyrate kinase, and Ack, respectively [65]. Homology searches using complete genome sequences revealed that, apart from phosphoglycerate kinase and *Pg*Ack, homologs of these enzymes are not present in *P. gingivalis* ATCC 33277 or W83 [24,25]. A homolog of phosphoglycerate kinase, one of the major enzymes in glycolysis, is present in the genome of *P. gingivalis* ATCC 33277 (PGN_0433). Previous experiments using radio-labeled glucose revealed only 5% uptake of glucose from the test medium by *P. gingivalis*, most of which contributes to cell carbohydrates and their derivatives, suggesting that glucose utilization by this organism is very poor, and carbohydrates do not appear to readily support growth [7]. Therefore, the PGN_0433 protein might not necessarily function as a phosphoglycerate kinase. In addition, apart from *Pg*Ack, no other analogs were identified in the genome of *P. gingivalis* ATCC 33277 or W83. Further studies are therefore needed to clarify the exact mechanism of ATP production in *P. gingivalis*, but the essentiality of both *Pg*Pta and *Pg*Ack implies that the Pta-Ack pathway plays an important role in ATP production in this microorganism.
