# Supplementary material for: Characterization of the phosphotransacetylase-acetate kinase pathway for ATP production in Porphyromonas gingivalis
Source: J Oral Microbiol. 2019 Apr 4;11(1):1588086. doi: 10.1080/20002297.2019.1588086 (PMC6461089; doi:10.1080/20002297.2019.1588086)
Supplement: Supplemental Material [file ZJOM_A_1588086_SM4258.zip › ZJOM_A_1588086/Supplemental Figures_rev2.pdf]

```
                  10        20        30        40        50        60        70        80
                   |         |         |         |         |         |         |         |
PgPta   -MDLIQDVIRRAQENKQRIVLPEGLEPRTLEAADRLMADKVVNIILIGNVDSVKAKVAELGLKNLDEAVIIDPNNHPKKQQ
MtPta   MVTFLEKISERAKKLNKTIALPETEDIRTLQAAAKILERGIADIVLVGNEADIKALAGDLDLS---KAKIVDPKTYEKKDE

                 90        100       110       120       130       140       150       160
                *|  *        |         |         |         |    *    |         *         |
PgPta    YTDLLLQIRQKKGLTPEKAAELVENPLYLGCLIVKSGDADGLIAGAQNTTGDVLRPALQVIKTAPGMTSVSGTFLLFTKA
MtPta    YINAFYELRKHKGITLENAAEIMSDYVYFAVMMAKLGEVDGVVSGAAHSSSDTLRPAVQIVKTAKGAALASAFFIISVPD

                170       180       190       200       210       220       230       240
                  |   *     |         |         |         |         |         |         |
PgPta    KEYGKDGLLLVADCAVIPNPTADELAQIAVATARTAKAIADIEPRVAMLSFSTKGSAKHEMTDKVVEATRMAQEMAPDLL
MtPta    CEYGSDGTFLFADSGMVEMPSVEDVANIAVISAKTFELLVQDVPKVAMLSYSTKGSAKSKLTEATIASTKLAQELAPDIA

                250       260       270       280       290       300       310       320
                  |         |         |         |         |         |         |         |
PgPta    IDGEMQADAALVERVAALKAPGSNVAGKANVLVFPTLEVGNIAYKLVERLGHAEAVGPILQGMAAPVNDLSRGCSVEDIY
MtPta    IDGELQVDAAIVPKVAASKAPGSPVAGKANVFIFPDLNCGNIAYKIAQRLAKAEAYGPITQGLAKPINDLSRGCSDEDIV

                330   336
                  |     |
PgPta    RMVAITANQAIAAKEQ
MtPta    GAVAITCVQAAAQDK-
```

Supplemental Figure S1. Sequence alignment of *Pg*Pta and *Mt*Pta. Conserved residues are shown with a gray background, and residues mutated in this study are shown with outline characters on a black background. Residue numbers for *Pg*Pta are indicated above the sequences. Asterisks indicate residues directly interacting with the bound acetyl-CoA. The boxed region corresponds to domain II.

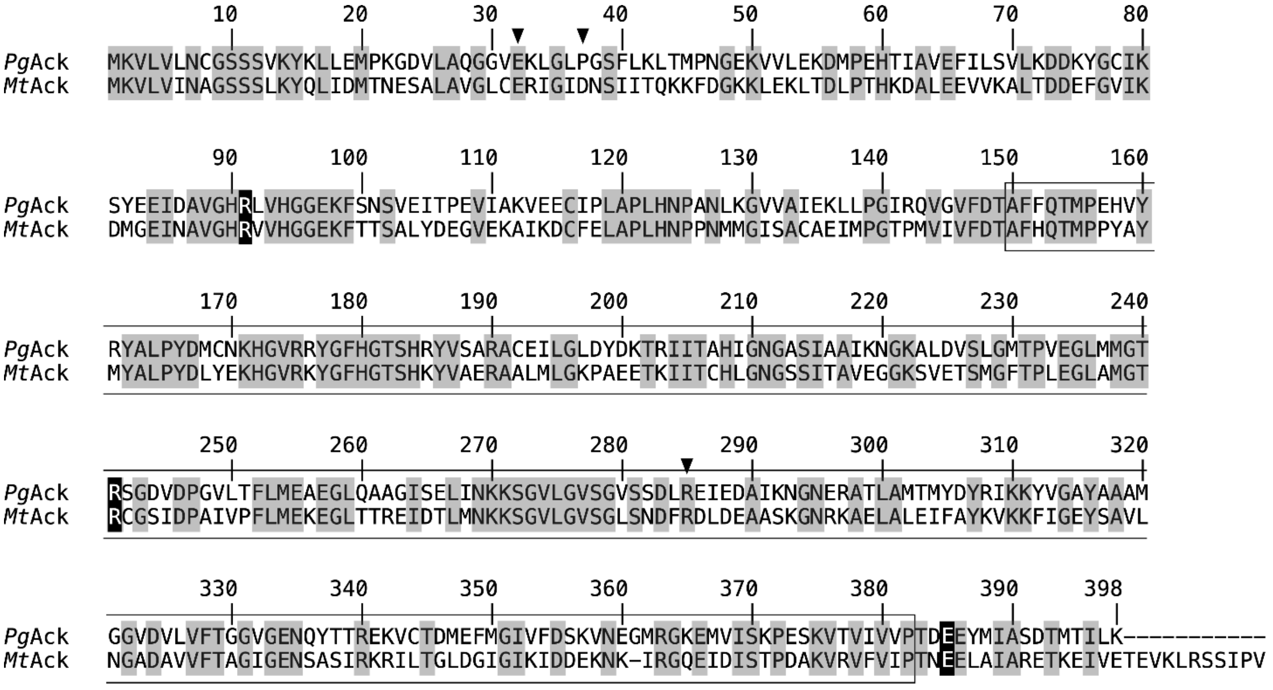

Supplemental Figure S2. Sequence alignment of *Pg*Ack and *Mt*Ack. Conserved residues are shown with a gray background, and residues mutated in this study are shown with outline characters on a black background. Residue numbers for *Pg*Ack are indicated above the sequences. Triangles indicate residues directly interacting between domains (Glu32 and Arg285) in the closed conformation (subunit D), and Pro37 located at the edge of the cleft. The boxed region corresponds to the C-terminal domain.

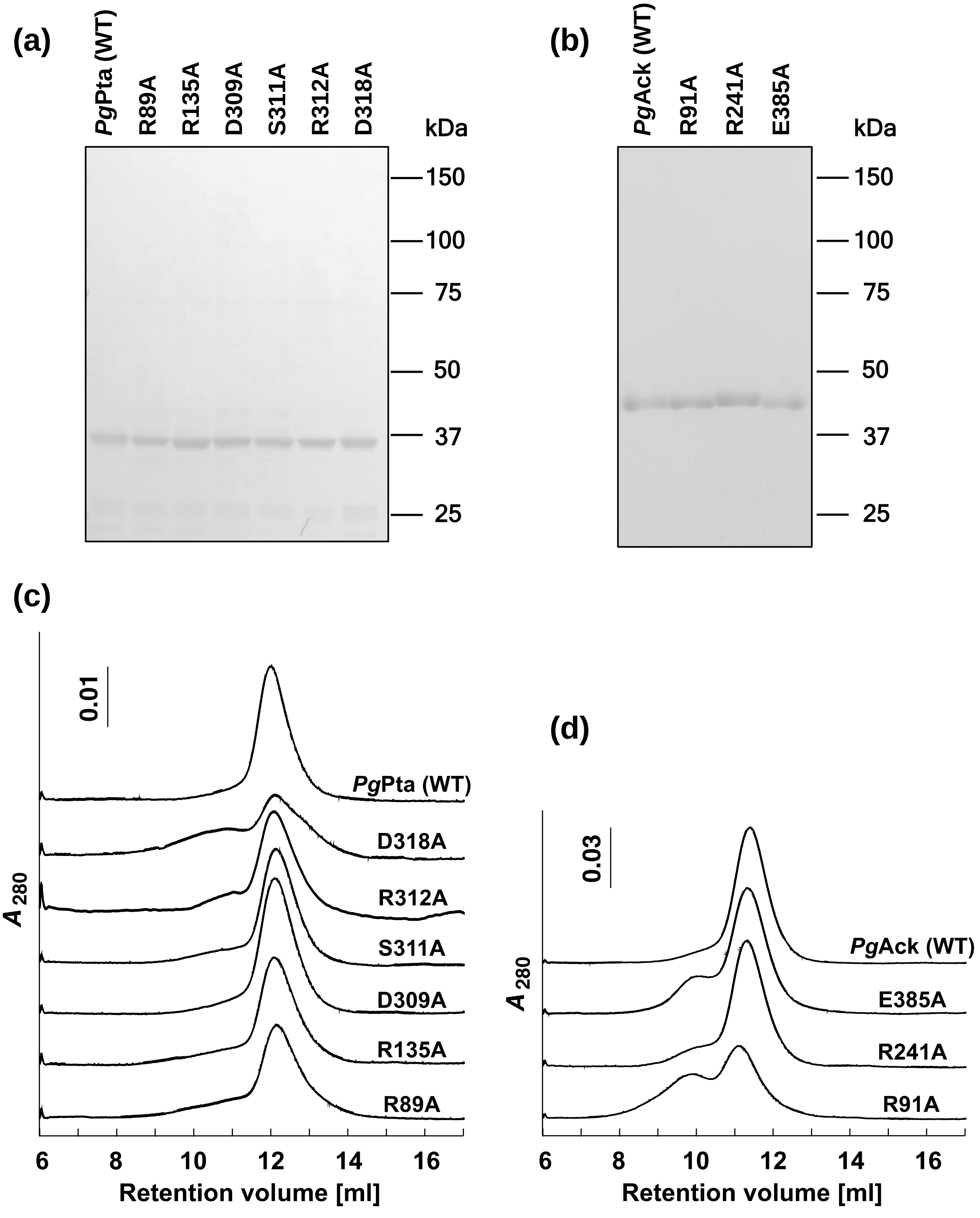

Supplemental Figure S3. Purification and gel filtration analysis of recombinant *Pg*Pta and *Pg*Ack mutant proteins**.** (a and b) SDS-PAGE analysis. Samples (~2 μg) were subjected to SDS-PAGE and visualized by Coomassie Brilliant Blue staining. The positions of molecular mass markers (in kDa) are shown. (c and d) Gel filtration analysis. Samples (60–240 μg/200 μl) were applied to a Superdex 200 HR 10/30 column equilibrated with PBS. The absorbance at 280 nm were normalized to the peak area of the WT protein.

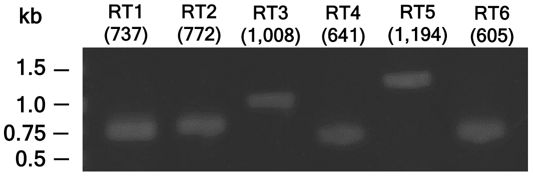

Supplemental Figure S4. Validation of primers used for RT-PCR. The genomic DNA of *P. gingivalis* ATCC 33277 was used as template. DNA size standards are shown.

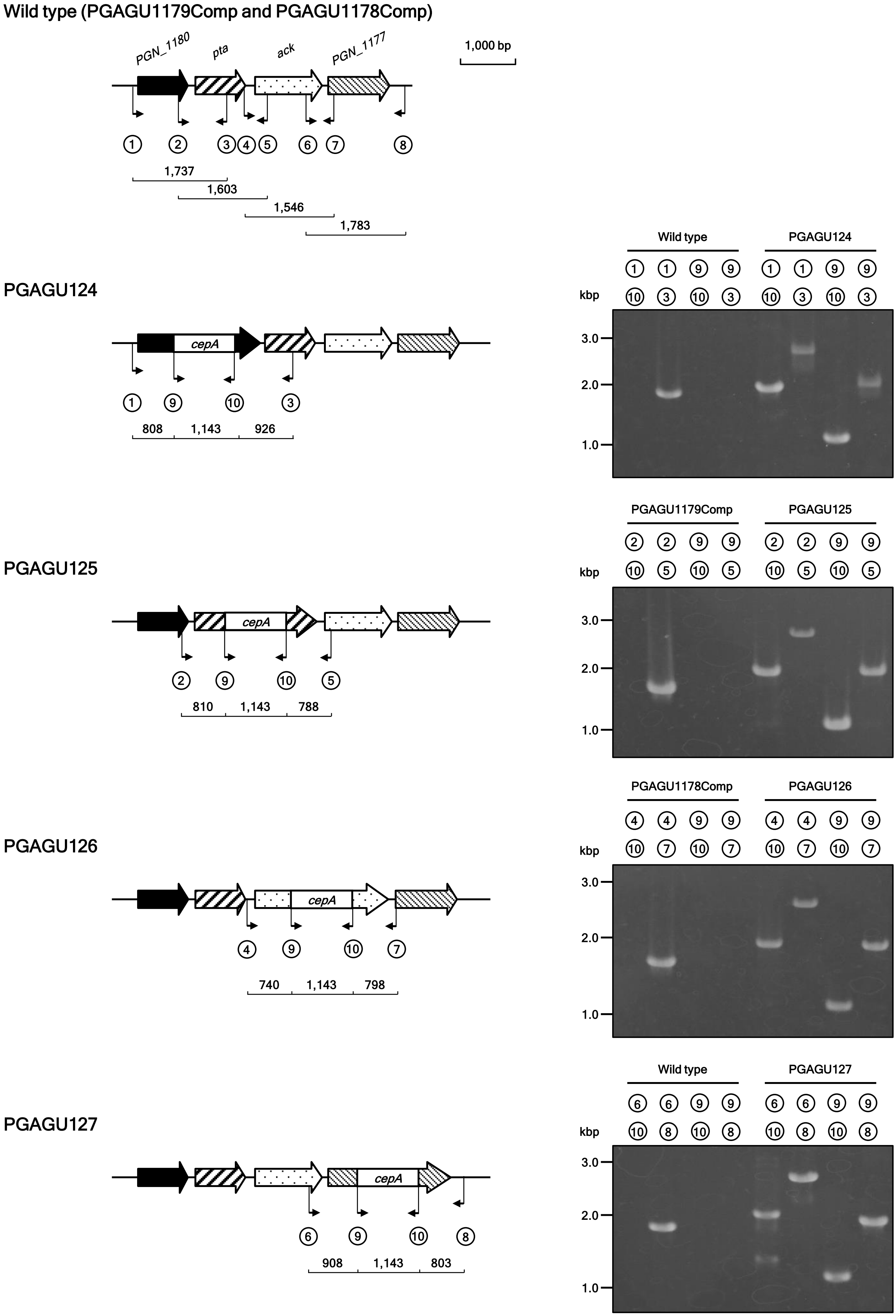

Supplemental Figure S5. Construction of *P. gingivalis* ATCC 33277 mutant strains. Chromosomal gene arrangement and verification by PCR analyses are shown. Each circled number indicates a PCR primer used for mutant verification. Distances between primers are given in bp. Each DNA fragment was PCR-amplified using the indicated primers. DNA size standards are shown.

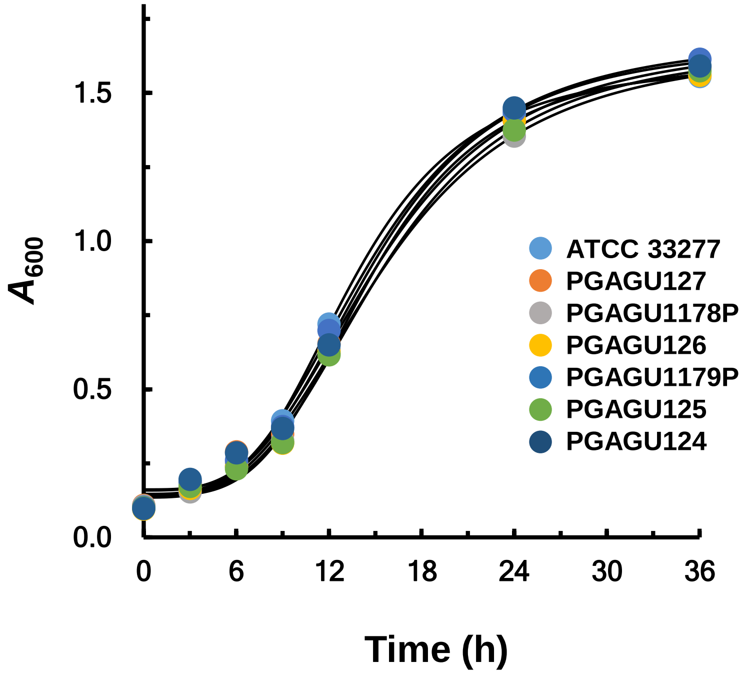

Supplemental Figure S6. A_{600} values of bacterial cell cultures.
