# Supplementary material for: Characterization of the phosphotransacetylase-acetate kinase pathway for ATP production in Porphyromonas gingivalis
Source: J Oral Microbiol. 2019 Apr 4;11(1):1588086. doi: 10.1080/20002297.2019.1588086 (PMC6461089; doi:10.1080/20002297.2019.1588086)
Supplement: Supplemental Material [file ZJOM_A_1588086_SM4258.zip › ZJOM_A_1588086/Supplemental Tables_rev.pdf]

Supplemental Table. Oligonucleotide primes used in this study.

Designation	Primer	Sequence (5' to 3')*
Confirmation of mutant strains		
cepA	120115-cepA-F	AAAAGAGTTAAGGAAAGTGAAGC
	120115-cepA-R	TTTCAAGTCACCGATAGTG
Upstream region of *PGN_1180*	060816-PGN1180-F1-MCL-Bam	TCTAGAACTAGTGGATCCAGAAGCTCTCAACGTGGTATG
	080916-PGN1180-R1-cep	GCTTCACTTTCCTTAACTCTTTTATCTTGTGATCGAGATTGCA
Downstream region of *PGN_1180*	080916-PGN1180-F2-cep	CACTATCGGTGACTTGAAATATCGTGGATGGGCAAAAG
	060816-PGN1180-R2-MCL-Sal	CCCCCCCTCGAGGTCGACACGTCTGATAACGTCTTGAATG
Upstream region of *pta*	060816-PGN1179-F1-MCL-Bam	TCTAGAACTAGTGGATCCTCTTTCGTCACGCATTAGATAG
	080916-PGN1179-R1-cep	GCTTCACTTTCCTTAACTCTTTTTTTGCCATATTCTTTGGCTT
Downstream region of *pta*	080916-PGN1179-F2-cep	CACTATCGGTGACTTGAAAACTTTTGCTTGTTGCAGACTG
	060816-PGN1179-R2-MCL-Sal	CCCCCCCTCGAGGTCGACCAGCACCACTTTCTCACCA
Upstream region of *ack*	060816-PGN1178-F1-MCL-Bam	TCTAGAACTAGTGGATCCGCCAAGGAGCAATAATAAAGAA
	080916-PGN1178-R1-cep	GCTTCACTTTCCTTAACTCTTTTAGCTCCATTGCCGATATGT
Downstream region of *ack*	080916-PGN1178-F2-cep	CACTATCGGTGACTTGAAATGCAATCAAAAACGGCAA
	060816-PGN1178-R2-MCL-Sal	CCCCCCCTCGAGGTCGACGGGGATTTACAACTACTTTC

		ACTC
Upstream region of *PGN_1177*	060816-PGN1177-F1-MCL-Bam	TCTAGAACTAGTGGATCCTGACACCGAAACGGCATAAC
	080916-PGN1177-R1-cep	GCTTCACTTTCCTTAACTCTTTTGAAGCATTACGATTGAAGTCG
Downstream region of *PGN_1177*	080916-PGN1177-F2-cep	CACTATCGGTGACTTGAAAGGGAAAGTTGTTTGTTAAACTGC
	060816-PGN1177-R2-MCL-Sal	CCCCCCCTCGAGGTCGACGCAAGATGGTTCGTGCAGGACAGC
Confirmation of mutant strains		
PGN_1180	111716PGN1180ko-checkF	TATGCCGTGGTTGACTTCTG
	111716PGN1180ko-checkR	AGCCTCGTCCAAGTTCTTCAA
Pgpta	112116PGN1179ko-checkF	ATAGAATACCTGTCCGTATCAGGA
	112116PGN1179ko-checkR	ATCCGTATTTGTCATCTTTGAG
Pgack	112116PGN1178ko-checkF	TATACAGGATGGTTGCCATCAC
	112116PGN1178ko-checkR	TTCTTGAAAGCATCGCCTT
PGN_1177	111716PGN1177ko-checkF	AAGGTGAATGAAGGTATGCGC
	111716PGN1177ko-checkR	TCTGTCTGGCTATCATGGAGGTA
Construction of recombinant proteins		
*Pg*Pta	PGN1179-F1-Bam	**AAGGATCC**GATCTCATTCAAGACGTTATCAGA
	PGN1179-R1-Sal	**AAGTCGAC**TTATTGCTCCTTGGCTGC
*Pg*Ack	pgn1178_NheI_F	**GGGGGGCTAGC**AAAGTATTGGTATTGAACTGTGGTAGTTC
	pgn1178_EcoRI_R	**GGGGGGAATTC**TTATTTGAGGATTGTCATGGTGTCGG

RT-PCR		
RT-1	010217-PGN1180F	AAGAAACGAAATCCATTCAAC
	010217-PGN1180R	GATACGGACAGGTATTCTATTGG
RT-2	010217-PGN1180-79F	GTAAGAATATTTGCAGGGAAAAAG
	010217-PGN1180-79R	CCTTTTTTTGGCGAATTTG
RT-3	011317-PGN1179-78F	GTGATCATTGATCCGAATAAC
	011317-PGN1179-78R	TGCCACTCTTTCTACCAAAG
RT-4	010217-PGN1179-78F	AGAGTGGCAGCACTCAAAG
	010217-PGN1179-78R	TTCTTCTACTTTGGCAATTACTTC
RT-5	PGN1178-F1-Bam	AAGGATCCAAAGTATTGGTATTGAACTGTGGTAG
	PGN1178-R1-Sal	AAGTCGACTTATTTGAGGATTGTCATGGTG
RT-6	010217-PGN1178-77F	GAGTCGATGTGCTCGTATTC
	010217-PGN1178-77R	GCAACGAAACAAAGCAATC
Construction of *Pg*Pta mutant enzymes		
R89A	11017-PGN1179-R89A-R	CCCTTTTTTTGGGCAATTTGC
	11017-PGN1179-R89A-F	GCAAATTGCCCAAAAAAAGGG
R135A	11017-PGN1179-R135A-R	GAAGAGCCGGAGCAAGTACATC
	11017-PGN1179-R135A-F	GATGTACTTGCTCCGGCTCTTC
D309A	11017-PGN1179-D309A-R	GCTCAAAGCGTTTACAGGTGCAG
	11017-PGN1179-D309A-F	CTGCACCTGTAAACGCTTTGAGC
S311A	11017-PGN1179-S311A-R	TCCGCGGGCCAAATCGTTTAC
	11017-PGN1179-S311A-F	GTAAACGATTTGGCCCGCGGA
R312A	11017-PGN1179-R312A-R	GAGCATCCGGCGCTCAAATC

	11017-PGN1179-R312A-F	GATTTGAGCGCCGGATGCTC
Construction of *Pg*Pta mutant enzymes		
R91A	pgn1178_R91A-F	GCGCTCGTTCATGGAGGTGAGAAGTTC
	pgn1178_R91A-R	GTGCCCTACTGCATCGATTTCTTC
R241A	pgn1178_R241A-F	GCGAGTGGAGACGTAGACCCCGG
	pgn1178_R241A-R	CGTACCCATCATAAGCCCTTCTAC
E385A	pgn1178_E385A-F	GCGGAATATATGATTGCCTCCGACACCATG
	pgn1178_E385A-R	GTCAGTGGGAACGACGATCACG

* Bold nucleotides indicate restriction endonuclease sites incorporated to facilitate cloning. Underlined nucleotides indicate an overlapping region of the cepA, or pMCL200.
